# Modulations of Foot and Ankle Frontal Kinematics for Breaking and Propulsive Movement Characteristics during Side-Step Cutting with Varying Midsole Thicknesses

**DOI:** 10.1155/2018/9171502

**Published:** 2018-05-09

**Authors:** Yi-Jia Lin, Shih-Chi Lee, Chao-Chin Chang, Tsung-Han Liu, Tzyy-Yuang Shiang, Wei-Chun Hsu

**Affiliations:** ^1^Graduate Institute of Biomedical Engineering, National Taiwan University of Science and Technology, Taipei, Taiwan; ^2^Department of Athletic Performance, National Taiwan Normal University, Taipei, Taiwan; ^3^National Defense Medical Center, Taipei, Taiwan

## Abstract

This study is aimed at determining the effects of midsole thickness on movement characteristic during side cutting movement. Fifteen athletes performed side-step cutting while wearing shoes with varying midsole thicknesses. Temporal-spatial and ground reaction force variables as well as foot and ankle frontal kinematics were used to describe breaking and propulsive movement characteristics and modulation strategies. Regardless of midsole thickness, temporal-spatial variables and breaking and propulsive force during side cutting were statistically unchanged. Significantly greater peaks of ankle inversion and plantarflexion with a thicker sole and greater midtarsal pronation with a thinner sole were observed. Current results demonstrated that hypotheses formed solely based on material testing were insufficient to understand the adaptations in human movement because of the redundancy of the neuromusculoskeletal system. Participants were able to maintain temporal-spatial performance during side cutting while wearing shoes with midsoles of varying thicknesses. Increased pronation for a thinner sole might help reduce the force of impact but might be associated with an increased risk of excessive stress on soft tissue. Increased peak of ankle inversion and plantarflexion for a thicker sole may be unfavorable for the stability of ankle joint. Information provided in human movement testing is crucial for understanding factors associated with movement characteristics and injury and should be considered in the future development of shoe design.

## 1. Introduction

Enhancement of sports performance and injury prevention possess both intrinsic and extrinsic factors. As footwear is an important extrinsic factor, researchers have examined the effect of certain footwear with specific design variables including material properties and thickness on lower limb muscle activities [1], proprioception, and stability [2]. Rather than using the traditional thick-sole design for its cushioning benefits, minimalist sports shoes have recently become a popular alternative [3–7]. In manufacturing, shoe minimalism can be facilitated by reducing the midsole thickness because the midsole layer is one of the crucial components of the shoe and often accounts for most of the volume and weight of a shoe. A thin midsole in minimalist sports shoes can compromise shock attenuation [6]. However, wearing a thick-midsole shoe creates a high foot position relative to the support surface, causing an unstable position [2]. Compared with being barefoot, the sole of a shoe increases the lever arm, thus contributing to increased external torque around the subtalar joint [8]. Theoretically, increasing midsole thickness can also amplify this leverage effect on external torque. Although numerous studies have demonstrated the influence of footwear on foot stability and injury, the independent effect of thickness from each element of a shoe component (i.e., upper, insert, midsole, and outsole) on foot and ankle joint mechanics remains unclear.

A challenge to determine the effect of footwear on human movement is the redundant nature of the musculoskeletal system, in which there are more joints and muscles that are necessary to accommodate the changing extrinsic conditions [9]. Athletes can achieve high level of temporal-spatial performance by one strategy while they can fail to maintain certain level of temporal-spatial performance because of another strategy possibly because some compensatory mechanism is essential for coping with the consequences of injuries. For example, researchers should account for the biomechanical role of high impact forces, rate of loading force, and pronation of the foot in injuries when considering the link between footwear design and sports injuries [10]. Unlocking of the transverse tarsal joint during pronation is crucial to reduce impact force, while excessive pronation has been recognized as a contributing factor for overuse running injuries and unfavorable for propulsion [11, 12]. As the biomechanics of the midfoot motions cannot be explained using a single-segment foot model, integrating biomechanical results into a shoe design requires sophisticated foot models to define the relative contributions of the middle and rear parts of the foot, which affect sports performance and injury risk [13–15]. However, few researchers have used multisegmented models to explore the relationship between shoe design and injuries and performance, which is necessary for describing foot kinematics within shoes [16, 17].

Movements involving a side cutting (SC) maneuver are critical during sports activities which can limit performance or induce risks of injuries [18]. Inverted ankle sprain, which frequently occurs during SC, can be induced by rapid ankle inversion, particularly when the ankle is in a plantar-flexed position [19]. One study concluded that appropriate shoe design can improve lateral stability of the ankle kinematics during SC movements [8]. Another one study reported decreases in ankle joint loading and increases in specific performance variables during SC movements when wearing 10 degrees of lateral banking incorporated in footwear, providing a protective mechanism without affecting performance for athletes [18].

The way that the neuromusculoskeletal system organizes the redundant degrees of freedom of the joints in order to maintain temporal-spatial performance during a side-cutting maneuver cannot be determined solely by pure material testing. Therefore, we investigated the effect of midsole thickness on temporal-spatial movement characteristics, ground reaction force (GRF) variables, and ankle and foot kinematics during lateral SC. We hypothesized the following: (a) Athletes who wear thin-soled shoes would demonstrate quicker temporal and greater spatial movement characteristics with higher propulsive force, which would enhance performance; however, high loading rate and breaking force caused by a thin sole would increase the risk of repetitive injuries. (b) Athletes who wear thick-soled shoes exhibit a greater midtarsal pronation range of motion during the early stance than do athletes who wear thin-soled shoes, which might strengthen shock absorption. In addition, athletes who wear thick-soled shoes demonstrate greater ankle inversion than do athletes who wear thin-soled shoes, which might be unfavorable for the joint position.

## 2. Materials and Methods

### 2.1. Subjects

Fifteen highly trained male badminton athletes (age: 21.37 ± 3.82 years) participated in this study. The athletes were all free from neuromusculoskeletal dysfunction, with normal or corrected vision. All participants were right-leg dominant, which was classified based on their preferred kicking leg. All athletes used their right hand to hold the badminton racket. Before data collection, written informed consent was obtained from each participant, and clearance to conduct the study was approved by the Institutional Human Research Ethics Committee.

### 2.2. Testing Shoes

The three testing badminton shoes contained the same vamp, shoe last, and insole. The midsole hardness of each shoe was 62 C (measured based on the Shore A scale), but the midsole thicknesses represented three distinct levels produced by the manufacturer (Victor Corporation): thin, medium, and thick. The three midsole thicknesses, 5 mm, 6 mm, and 7 mm, represented the range of thicknesses commonly used in current badminton footwear produced by the manufacturer. Testing order was randomized.

### 2.3. Data Collection

Passive infrared retroreflective markers were attached to the skin of body segments to determine their spatial positions, including the shank (head of the fibula (HF), tibial tuberosity (TT), and medial and lateral malleolus (MMA and LMA)), the rearfoot (upper central ridge of the calcaneus posterior surface, i.e., Achilles tendon attachment (CA), most of the medial apex of the sustentaculum tali (ST), and the lateral apex of the peroneal tubercle (PT)), and the midfoot (most of the medial apex of the tuberosity of the navicular (TN), the base of the fifth metatarsal (VMH), the dorsolateral aspect of the fifth metatarsocuboid joint (VMB), the base of the second metatarsal (SMH), and the dorsomedial aspect of the second metatarsocuneiform joint (SMB)). Motion data were measured using a motion analysis system equipped with ultra-high-resolution infrared cameras at a sampling rate of 200 Hz. To reconstruct the 3D coordinates of the markers from 2D video images, the Vicon system was calibrated using calibration objects. These objects were designed to allow the cameras to measure the motion of the markers with an accuracy within 1 mm. Calibrated force platforms (AMTI, Advanced Mechanical Technology, USA) with 2000 Hz sampling rates, connected to a multichannel charge amplifier, were embedded in the floor to measure the GRF produced during side cutting, which were also used to define movement cycle.

The participants were instructed to perform lateral side cutting while infrared-retroreflective markers with a diameter of 14 mm were used to track the motion of the shank and foot segments. Marker placement on shoes has previously been demonstrated to overestimate calcaneal movement during running. Therefore, shoe windows were constructed to allow placement of the markers directly on the bony landmarks of the calcaneus and midfoot [20]. Each participant was familiarized with the lateral SC task and then was asked to perform three successful trials.

**Figure 1:**
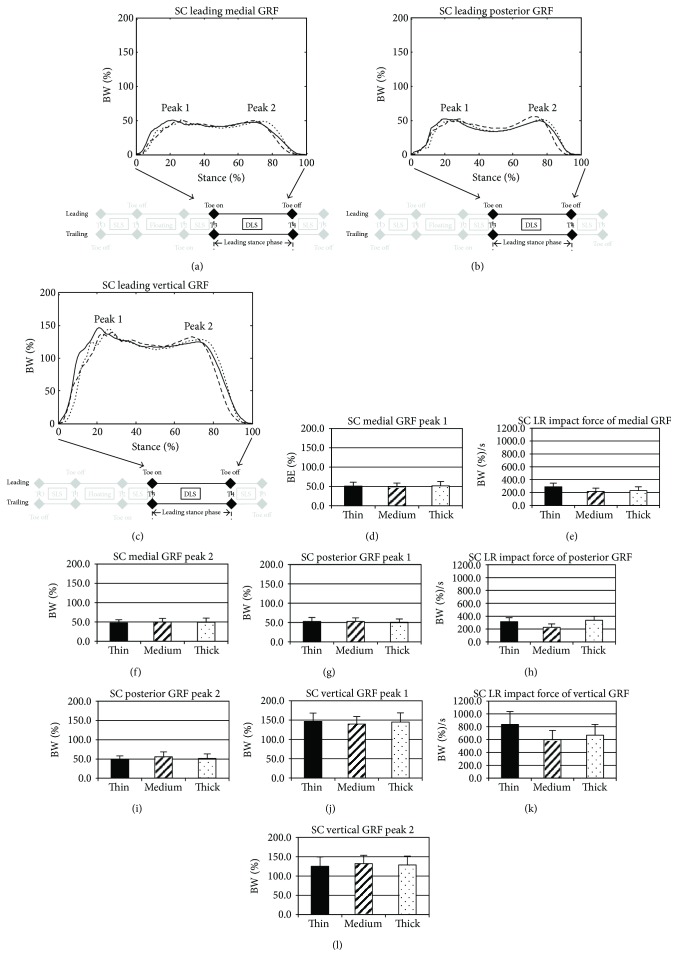
GRF curves on the (a) medial, (b) posterior, and (c) vertical directions of the leading stance limb when performing lateral SC for the thin (thick lines), medium (dash lines), and thick (dot lines) midsole during the stance phase. Peak 1 indicates the peak GRF during the early leading stance, and peak 2 indicates the peak GRF during the late leading stance. Comparisons of these peaks and the loading rate of impact peaks between midsole conditions are displayed as bar charts in (d)–(f) for the medial GRF, (g)–(i) for the posterior GRF, and (j)–(l) for the vertical GRF, with corresponding standard deviations as error bars (black bar: the thin midsole, dashed bar: the medium midsole, and dotted bar: the thick midsole).

The movement task performed in this study required the participants to perform SC footwork with right-limb leading. Therefore, the leg of the racket-hand side (right limb) was defined as the leading limb, and the leg opposite to the racket-hand side (left limb) was thus a trailing limb. The SC movement required the participants to perform lateral SC footwork with the lateral step leading the right limb toward the force platform. The SC involved five phases: the leading single-limb support (SLS) phase, the floating phase, the first trailing SLS phase, the double-limb support (DLS) phase, and the second trailing SLS phase. These phases were divided by six critical events: the trailing toe off, the leading toe off, the trailing toe on, the leading toe on, the leading toe off, and the trailing toe off (Figure 1). The effort at which the participants performed the task was standardized by equalizing the starting position relative to the force platform according to each player's familiar position and by positioning the ball at the same position and height.

### 2.4. Data Analysis

Temporal-spatial variables, including total, breaking, and propulsive impulse duration of the leading stance phase as well as the leading step length, and step time were calculated. The total impulse duration refers to the duration of the total stance phase, the passive impulsive duration refers to the duration from the beginning of the leading stance phase to the time when the vertical GRF decreases to its lowest value, and the active impulsive duration refers to the duration from the time when the vertical GRF decreases to its lowest value to the end of the leading stance phase [21].

For dynamic analysis during each of the movement trials, the shank, rearfoot, and midfoot were modeled as a three-link system. Each link was embedded with an orthogonal coordinate system. Orientation of the axes, *x*-axis pointing forward, *y*-axis pointing upward, and *z*-axis pointing to the right, was based on ISB recommendation [22].

Ankle joint kinematic calculations were based on the virtual rearfoot referenced to the shank segment. Midtarsal joint kinematic calculations were based on the virtual midfoot referenced to the rearfoot segment, where the shank local coordinate system, the rearfoot local coordinate system, and the midfoot local coordinate system were defined as follows: For the shank local coordinate system, the origin point, *O*_sha_, is located at the middle point between LMA and MMA, the *X*_sha_-axis is orthogonal to the frontal plane defined by LMA, MMA, and HF, the *Z*_sha_-axis is orthogonal to the sagittal plane defined by the *X*_sha_-axis and TT, and the *Y*_sha_-axis is orthogonal to the *XZ*_sha_ plane (transverse plane). For the rearfoot local coordinate system, the origin point is at CA, the *X*_rear_-axis direction is defined by CA and the middle point, IC, between ST and PT, the *Y*_rear_-axis is orthogonal to the transverse plane defined by the *X*_rear_-axis and ST, and the *Z*_rear_-axis is orthogonal to the *XY*_rear_ plane (sagittal plane). For the midfoot local coordinate system, the origin point, *O*_mid_, is at the middle point between TN and VMB, the *X*_mid_-axis direction is defined by the *O*_mid_ and SMB, the *Y*_mid_-axis is orthogonal to the transverse plane defined by the *X*_mid_-axis and TN, and the *Z*_mid_-axis is orthogonal to the *XY*_mid_ plane (sagittal plane).

The definitions and terms for the description of the rotational movements of the joints using a Cardanic rotation sequence (*z*–*y*–*x*) were according to the ISB recommendation [22]. In the recommendation, the rotational movements of the joint are described by motions about three anatomical axes. More specifically for the ankle joint, the dorsi-/plantarflexion is the rotation about the *z*-axis of the proximal shank segment, the eversion/inversion is about the *x*-axis of the distal rearfoot segment, and the internal/external rotation is about the axis orthogonal to the *z*-axis of the proximal segment and the *x*-axis of the rearfoot segment (floating axis); also, for the midtarsal joint, the supination/pronation is about the *x*-axis of the distal midfoot segment [23].

The kinematic variables analyzed during the task were the values of the frontal plane joint angle of the ankle (inversion and plantarflexion) and midtarsal joints (pronation). The patterns of angular displacement of the ankle and midfoot joint during the cutting movements were provided to have a clear illustration of the key concerns discussed in our study. The leading stance phase which comprised the DLS of a complete side-cutting maneuver will be taken as the time period during which key variables are analyzed. Ankle inversion at the beginning and the end of the leading stance phase (the DLS phase of the complete side cutting), the peak values of the frontal plane joint angle of the ankle (inversion) and midfoot (pronation) during the DLS, and the loading rate of impact peak and the peak of the GRFs in the medial, posterior, and vertical directions were extracted. The mean values of each calculated variable for the entire group of participants were obtained.

### 2.5. Statistical Analysis

To analyze thickness effects, comparison of the calculated variables between shoes with three midsole thicknesses was performed using a nonparametric repeated-measure analysis of variance by using SPSS 19.0 (SPSS Inc., USA). When a thickness effect was observed, a post hoc analysis was performed using a polynomial test to determine the trend (linear or quadratic). The level of significance was set at *α* = 0.05.

## 3. Results

Based on the observation of the participants performing lateral SC, no significant differences in the total stance phase, passive breaking impulsive phase, active propulsive impulsive phase duration, leading step time, or step length were observed among the various midsole thicknesses (*p* > 0.05, Table 1). The subjects were able to maintain constant temporal-spatial performance regardless of midsole thickness. The magnitude of the GRF for each participant was displayed for the medial (Figure 1(a)), posterior (Figure 1(b)), and vertical (Figure 1(c)) directions of the leading stance limb. No significant thickness effects were observed for the early impact peaks (Figures 1(d), 1(g), and 1(j)) or the loading rates (Figures 1(e), 1(h), and 1(k)) (*p* > 0.05). No significant thickness effects were discovered for the late impulsive peaks (Figures 1(f), 1(i), and 1(l)) (*p* > 0.05).

**Figure 2:**
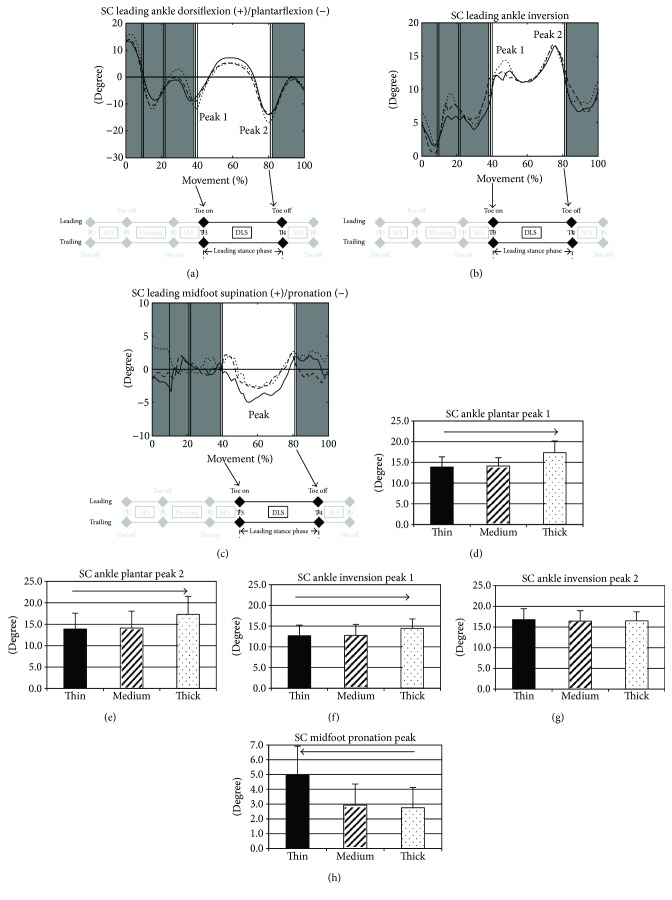
Angular displacement curves of the (a) ankle dorsiflexion (+)/plantarflexion (−), (b) ankle inversion, and (c) midfoot supination (+)/pronation (−) GRF of the leading stance limb when performing lateral side cutting for the thin (thick lines), medium (dash lines), and thick (dot lines) midsoles during the stance phase. The vertical lines indicate the instances when the leading toe is off (T1), the trailing toe is on (T2), the leading heel strikes (T3), and the leading heel is off (T4) the ground. Focusing on the leading stance phase, peak 1 indicates the peak ankle plantarflexion and peak ankle inversion during the early leading stance phase and peak 2 indicates the peak ankle plantarflexion and peak ankle inversion during the late leading stance phase and the “peak” midfoot pronation around the middle of leading stance phases. Comparisons of these peaks among midsole conditions are displayed as bar charts in (d) and (e) for ankle plantarflexion, (f) and (g) for ankle inversion, and (h) for midfoot pronation, with corresponding standard deviations as error bars. With increasing midsole thickness, the right arrow above the graph indicates a statistically significant increasing linear trend and the left arrow indicates a statistically significant decreasing linear trend (*p* < 0.05) (black bar: the thin midsole, dashed bar: the medium midsole, and dotted bar: the thick midsole).

A significant thickness effect was observed for the frontal plane angles of the ankle joints at initial contact of the leading toe (T3) and at the leading toe off (T4) during lateral SC movement. An increase in midsole thickness exhibited linearly significant difference with ankle inversion (Table 1). A significant thickness effect was observed for the early and late peak plantarflexion (Figures 2(d) and 2(e)) and the early peak inversion (Figures 2(f) and 2(g)) during the leading stance phase (*p* < 0.05). For frontal plane midfoot kinematics, a significant thickness effect was observed for the peak midtarsal joint pronation during weight acceptance of the leading limb (Figure 2(h), *p* < 0.05).

## 4. Discussion

We investigated the effects of midsole thickness on temporal-spatial movement characteristics, GRF variables, and ankle and foot kinematics during lateral SC. We observed similar temporal-spatial performance among shoes with various midsole thicknesses, which might indicate the ability to maintain aspects of side-step agility despite the difference in midsole thickness. To maintain temporal-spatial performance, similar passive breaking impulse duration, active propulsive impulse duration, total stance duration, and leading step time (Table 1) were able to facilitate quick-cutting movements performed by the badminton athletes even they were wearing shoes with various midsole thicknesses. Similar leading-step length measurements also suggested that athletes were able to maintain spatial performance regardless of the level of midsole thickness (Table 1).

Movements involving an SC maneuver placed the participants' feet into an unfavorable position for side-to-side joint stability. Previous studies have provided information about the GRF characteristics of cutting movements in healthy players and in players with ankle instability [24]. In addition to GRF variables, our study provided kinematic evidence supporting the risk of lateral ankle sprain during cutting movements. The statistical significance of increased ankle inversion and plantarflexion (at initial contact of the leading limb and at the two peaks during the leading stance phase) indicated this relatively unstable position (Table 1 and Figure 2), which suggested that players with functional instability of the ankle joint might be at risk of onset or recurrent ankle sprain if they wear thick-midsole shoes. However, current kinematic evidence associating thick midsoles with a risk of lateral ankle sprain may not be sufficient because the recorded inversion angles were approximately 17°, which was lower than the suggested threshold of about 40° required to identify an ankle sprain injury hazard [25, 26]. Lateral ankle sprain is the most common badminton injury, which commonly occurs with plantarflexion and inversion [27]. Although no previous studies have targeted the effect of midsole thickness on dynamic sports activities, one study reported that plantarflexion and inversion angles are underestimated more than dorsiflexion and eversion angles are under both thick- and thin-sole shoe conditions, compared with being barefoot [28]. Therefore, footwear designers should focus on preventing placement of the foot in an inverted position. Our ankle kinematic results corresponded closely with those of previous studies, which have reported that wearing a thick-midsole shoe causes a high foot position relative to the support surface and enhances the effect of leverage on external torque, thereby creating an unstable position [1]. The amplitude of oscillatory movements might increase for a high foot position because of thick-midsole shoes; displacing the center of the foot mass might affect the ability to adjust posture sufficiently. Still, we proposed that the effect of thickness on ankle joint mechanics might produce an increased risk of lateral ankle sprain. High-top shoes have been recommended for reducing the risk of ankle sprains by limiting ankle inversion [29]. A high shoe cut combined with a thick midsole might be recommended for middle-aged or older patients, who require a higher level of protection than of agility during exercise. However, studies of shoes for basketball players have failed to provide convincing evidence for the ability of high-top shoes to prevent ankle injuries. Considering the varying conclusions on the usefulness of high-top shoes for increasing ankle stability and the acceptance by athletes, maintaining flexibility in the sagittal plane at both the ankle and metatarsophalangeal joints may be another option, thereby reducing the risk of ankle injury for players who wear thick-midsole shoes.

In addition to deviated ankle mechanics during weight acceptance of lateral SC movements for the thick-midsole condition, we observed greater pronation at the midtarsal joint during lateral SC for thin-midsole shoes than for thick-midsole shoes (Figure 2(h)). In theory, a thin sole deforms less than a thick sole does, and impact forces are less attenuated. Greater loading rate was associated with an increased risk of stress fractures and plantar fasciitis in runners [30]. However, according to our GRF data, no significant thickness effect existed on GRF peaks and the loading rate (Figure 1), which might be contributed by the observed compensatory increased pronated motion at the midtarsal joint during lateral SC with thin-midsole shoes (Figures 2(c) and 2(h)), because pronation during stance is a good modulation to dissipate stress [31]. The increased pronation observed under the thin-midsole condition in this study may be a protective mechanism to attenuate impact forces. However, repetitive highly pronated motion might cause adverse effects on foot mechanics. Shoe designers should consider antipronation in a thin-midsole series to increase midfoot stability when playing sports that require intensive footwork involving SC.

Although we controlled the material properties, including hardness and elasticity, to eliminate factors other than thickness that might have affected the results, increased thickness of the same material can cause a reduction in stiffness, which might cause increased shear movement of the midsole during lateral movements; this is a potential limitation of studies that changes the geometry of one component of shoes without reporting other possible changes in physical characteristics of the shoes that are caused by the changes of the shoes' geometry. Researchers may also further examine the functional concept of the midsole in sports footwear by analyzing the kinematic strategies and joint mechanics of hip joints, knee joints, and the trunk. In addition, this study reports the effects of midsole thickness for side cutting maneuver; it remains unknown to what extent, if any, a preparatory strengthening program targeting on the stability of ankle and midtarsal joint would be incorporated while selecting shoes with midsoles of varying thicknesses.

## 5. Conclusion

Athletes who wear thinner-soled shoes exhibit a greater midtarsal pronation than do athletes who wear thicker-soled shoes, which might strengthen shock absorption. However, repetitive functional adaptation to the thin sole revealed by the increased pronation might alter foot structure and, consequently, kinesiology, in the long term. Athletes who wear thick-soled shoes demonstrate greater ankle inversion during early weight acceptance than do athletes who wear thin-soled shoes, which might be unfavorable for ankle-joint stability. Biomechanical advantages formed based on pure material testing related to thickness of midsoles were incapable of fully accounting for adaptations in human movement. By studying human movement, researchers can better clarify how footwear influences the biomechanical factors that can possibly improve sports performance or lead to injury and understand the implications of these results for advertisement in the footwear market.

## Figures and Tables

**Table 1:** Temporal-spatial variables and ankle inversion at each of the critical events during side cutting movement for the three midsole thicknesses (thin, medium, and thick).

		Thickness			Thickness effects
		Thin	Medium	Thick	*p*
Temporal variables	Total leading stance duration (sec)	0.63 ± 0.13	0.60 ± 0.15	0.62 ± 0.15	0.15
	Breaking impulse duration of the leading stance phase (sec)	0.33 ± 0.13	0.30 ± 0.14	0.32 ± 0.12	0.28
	Propulsive impulse duration of the leading stance phase (sec)	0.31 ± 0.08	0.31 ± 0.07	0.31 ± 0.07	0.92
	Leading step time (sec)	0.53 ± 0.07	0.55 ± 0.07	0.54 ± 0.07	0.41

Spatial variables	Leading step length (cm)	138.89 ± 25.19	145.67 ± 23.31	141.31 ± 26.96	0.31

Ankle inversion	At the beginning of the leading stance phase	7.82 ± 5.82	8.91 ± 4.92	11.82 ± 6.55	0.016^∗^
	At the end of the leading stance phase	10.37 ± 5.28	11.73 ± 4.81	16.27 ± 5.36	0.013^∗^

*Note*. Values are expressed as means ± SD. *p* values for comparisons between thin, medium, and thick midsole conditions. ^∗^*p* < 0.05 indicates a significant difference.
